# High Colonization Possibility of Some Species of Weeds in *Suaeda salsa* Community: From an Ecological Stoichiometry Perspective

**DOI:** 10.1371/journal.pone.0170401

**Published:** 2017-01-30

**Authors:** Changzi Ge, Renqing Wang, Yanchao Chai, Haiqing Wang, Manman Kan, Jian Liu

**Affiliations:** 1 Institute of Ecology and Biodiversity, School of Life Sciences, Shandong University, Jinan, China; 2 Marine College, Shandong University, Weihai, China; 3 Institute of Environmental Research, Shandong University, Jinan, China; Chinese Academy of Forestry, CHINA

## Abstract

*Suaeda salsa* community is a vegetation type in saline-alkali areas. Weed invasion and colonization in *S*. *salsa* communities lead to fragmentationsof *S*. *salsa* communities. The colonization of invaded weeds in *S*. *salsa* communities is related to community succession of saline-alkali zones. The fragmented *S*. *salsa* community may be restored if the mechanism of invaded weed colonization in *S*. *salsa* communities is clearly elucidated. Thus, we studied the ecological stoichiometric characteristics of soils and plants in a salt marsh to explain the high colonization possibility of invaded weeds in *S*. *salsa* communities. In October 2014, soils and plants were collected from Dongfeng Salt Marsh, Jiaozhou Bay, Shandong Province, China. The ratio of Ex-N/Ex-P in soil was less than 13, which suggests a relative nitrogen limitation for the primary production in the zone. The minimum phosphorus content in plants was higher than 1 mg g^-1^, whereas the maximum nitrogen content in plants was less than 13 mg g^-1^. These results imply that phosphorus was abundant, whereas nitrogen was deficient in the area. The plants in the salt marsh may be limited by nitrogen. Given the relatively lower nitrogen contents in *Cyperus glomeratus*, *Echinochloa crusgalli*, and *Aster subulatus* than that in *S*. *salsa*, these three species exhibited higher competitiveness than S. salsa did when nitrogen was limited in primary production. These weed species may colonize highly in *S*. *salsa* communities. Moreover, nitrogen fertilization might be effective to maintain *S*. *salsa* community in Dongfeng Salt Marsh, whereas its effects on controlling weeds colonization in *S*. *salsa* communities need more studies to verify.

## Introduction

*Suaeda salsa* belongs to the family *Chenopodiaceae*, and this species can tolerate adverse environmental conditions, which enable them to survive in soils with high salinity or alkalinity [1]. *S*. *salsa* community is a typical dominant vegetation in saline-alkali areas (e.g., occurrences of large *S*. *salsa* communities in the Yellow River Delta and coastal areas of Jiangsu Province, China) [2–4]. Numerous *S*. *salsa* communities have been developed as tourism resources in saline-alkali areas. Aside from being important habitats of birds and invertebrates, *S*. *salsa* communities can regulate transportation or transformation of nutrients and heavy metals [5, 6]. Moreover, *S*. *salsa* may reduce the salinity and alkalinity in soils, and improve the organic matter contents or microbial species in soils [7, 8]. Thus, the *S*. *salsa* communities succession is important for saline-alkali areas. Invasion of some species of weeds are common in *S*. *salsa* communities [9]. After colonization of invaded weeds, *S*. *salsa* communities become fragmented, which may influence their succession and ecological values. At suitable conditions, *S*. *salsa* community may be replaced by *S*. *salsa*+ *Polygonum sibiricum* community [10], *S*. *salsa*+ *Phragmites australis* community [11], *Aeluropus sinensis* or *Imperata cylindrica* community [12]. Why can these invaded weeds colonize *S*. *salsa* communities? Do these fragmentations of *S*. *salsa* communities imply the succession trend of *S*. *salsa* communities? How can the colonization of invaded weeds be prevented in *S*. *salsa* communities? These questions are related not only to inter-specific competition between *S*. *salsa* and invaded weeds, but also to community succession in saline-alkali zones. The superior photosynthetic characteristics and higher energy utilization efficiency contribute the successful invasions of *Mikania micrantha*, *Ageratina adenophora*, *Chromolaena odorata* and *Bidens pilosa* to native communities [13]. Nevertheless, *Ipomoea cairica* may change nutrient contents ratio of soil to enhance its invasion abilities [14]. *Solidago canadensis* invades in the special environment of riparian zone successfully by its ability of absorption and accumulation nitrogen and phosphorus [15]. Almost these studies are related to alien invasive plants invasion mechanisms, whereas most weeds colonized in *S*. *salsa* communities belong to native species. Moreover, data to address mechanisms of invasion and colonization of weeds in *S*. *salsa* communities are scarce.

Community structure and community succession may be influenced by nutrients stoichiometry characteristics [16–19], hence, it's assumed that invasion and colonization of weeds in *S*. *salsa* communities are driven by nutrients stoichiometry characteristics. The present work aims to explain the high colonization possibility of some species of weeds in *S*. *salsa* communities from a stoichiometric perspective. The results may provide possible applications of ecological stoichimetry for community succession assessment and determination regulation methods to speed up or slow down *S*. *salsa* community succession in saline-alkali areas.

## Materials and Methods

### Materials

In October 2014, soils and plants were sampled in Dongfeng Salt Marsh (36°02′N, 120°13′E) located in the northern part of Jiaozhou Bay, Qingdao, Shandong Province, China. The sampling site is an abandoned saltern, which has a history of 100 years and it was abandoned from 1996. It belongs to Qingdao Dongfeng Saltworks and its owner gave permission to conduct the study because the vegetation wouldn't be destroyed and there is no endangered or protected species in the area.

The typical vegetation in the salt marsh is *S*. *salsa* community, and the dominant phenotype of *S*. *salsa* is red. *S*. *salsa* can grow to heights of 20 to 40 cm.*S*. *salsa* becomes into flowering and fruit periods in the salt marsh from August to October. Some species of *Gramineous* (such as *Setaria viridis* and *Echinochloa crusgalli*), *Cyperaceae* (such as *Cyperus glomeratus*), *Compositae* (such as *Aster subulatus*), *Polygonaceae* (such as *Polygonum aviculare*), and *Chenopodiaceae* (such as *Suaeda glauca*) have colonized in *S*. *salsa* communities in the area. The invaded *S*. *salsa* communities become fragmented.*A*. *subulatus*, *E*. *crusgalli*, *P*. *aviculare* and *S*. *viridas* are on the list of alien invasive species in China, and the invasive grade of *A*. *subulatus* is 1 and the invasive grades of other weeds are 7, which means that *A*. *subulatus* belongs to malignant invasive species and others become Chinese domestic species [20].

**Fig 1:**
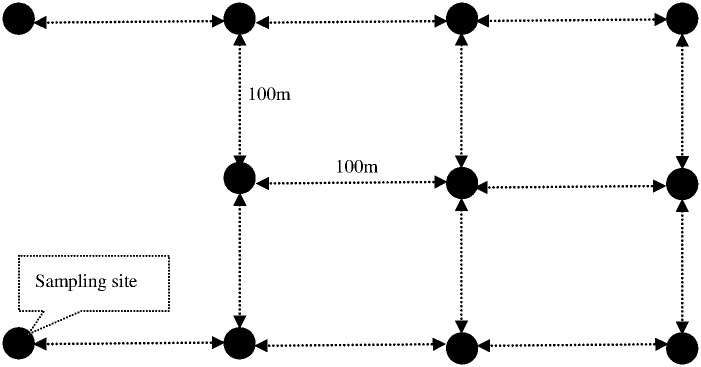
Sketch map of sampling sites in Dongfeng Salt Marsh.

Surface soils with a depth of 20 cm under vegetation, plants were sampled in 11 randomly designed sampling sites by a 1 m ×1 m quadrat (Fig 1). The replicates for selection in plants and soils in every site was 3. Based on the soil surface level, the plants were differentiated over and under-ground parts. Moreover, the vegetations in the 1st, 3rd, 5th, 6th, 8th and 10th sampling site were *S*. *salsa* communities. The vegetation in the 2nd, 4th, 7th, 9th and 11th sampling site was *S*. *salsa*+ *S*. *viridis* community, *S*. *salsa*+ *C*. *glomeratus*+ *A*. *subulatus*+ *E*. *crusgalli* community, *S*. *salsa* + *P*. *aviculare*+ *S*. *glauca* community, *S*. *salsa* + *A*. *subulatus* community and *S*. *salsa* + *P*. *aviculare* community, respectively.

### Measurements

The plants were dried to constant weight at 65°C, and the dried plant samples were crushed using Chinese medicine grinder. The nitrogen content in plants (calculated by the dry weight of the plant) was measured with an elemental analyzer (Elemental Analyzer Vario EL). To measure phosphorus content in plants (calculated by the dry weight of the plant), we treated the dried plants through the wet oxidation method, in which the reagents were concentrated sulfuric acid (H_2_SO_4_) and hydrogen peroxide (H_2_O_2_) solution. The dried plant organs with weight of 0.1 g and 5 mL H_2_SO_4_ were put into one beaker. After 12 hour, H_2_O_2_ was added into the beaker gradually to make the mixture limpid. Then, it was diluted to determine the phosphorus content by the phosphomolybdate blue method.

As the plants roots in soils were collected and screened, the soil samples were dried to constant weight at 65°C. The water content in soil was measured through the dehydration method and calculated by $\frac{100(w_{wet}-w_{65})}{w_{wet}}$, where *w*_*wet*_ and *w*_65_ wasthe weight of wet soil (g) and weight of dried soil at 65°C (g), respectively. The dried soil samples were burned at 450°C for about 5.5 h to determine the organic matter content in soil. The organic matter content was calculated by $\frac{100(w_{65}-w_{450})}{w_{65}}$, where *w*_450_ was the ash weight of burned soil at 450°C (g). The dried soil with weight of 0.10 g was added into 25 mL of oxygenant that consisted of K_2_S_2_O_8_ (0.15 mol/L) and NaOH (0.15 mol/L). After 1-h nitration at 120°C and 0.12 MPa, the mixture was centrifuged at 4000 r/min for 10 min. The supernatant was collected to determine the total phosphorus (TP) content and the total nitrogen (TN) content, which were both calculated using the dry weight of soil. Dried soil (1.00 g) was added into 25 mL of MgCl_2_ (1 mol/L). After 2-h oscillation, the mixture was centrifuged at 4000 r/min for 10 min. The supernatant was collected to determine the exchangeable phosphorus (Ex-P) content and the exchangeable nitrogen (Ex-N) content, which were both calculated using the dry weight of soil. Moreover, the Ex-N included nitrate, nitrite and ammonia nitrogen.

### Statistical analysis

If the homogeneity of variances was satisfied, one-way ANOVA was conducted to analyze the difference in nitrogen and phosphorus contents in different parts or species of plants, as well as difference in organic matter contents in soil in different sampling sites. Multiple comparisons were conducted through the Bonferroni-test. Otherwise, the difference was determined through the Dunnett’s-test. The significant level was 0.05.

## Results

### Matter contents in soil

**Fig 2:**
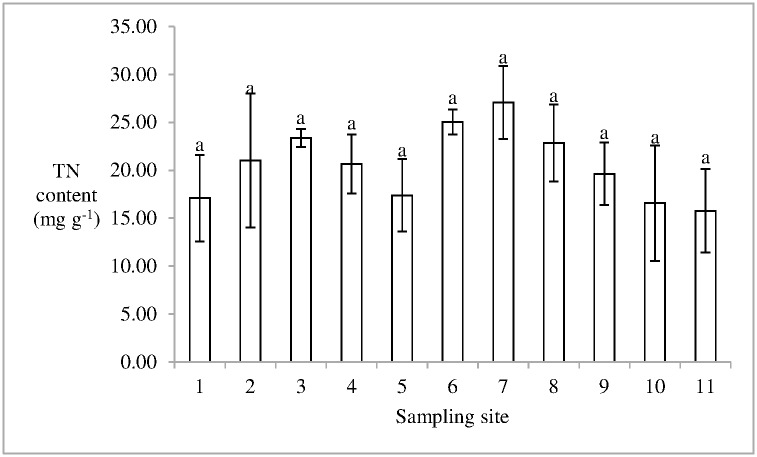
TN content in soil in Dongfeng Salt Marsh. Different litters indicate the statistical differences (p < 0.05).

**Fig 3:**
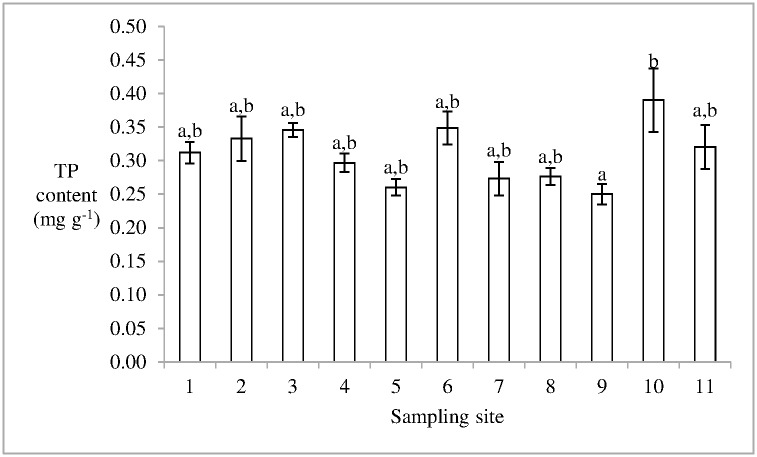
TP content in soil in Dongfeng Salt Marsh. Different litters indicate the statistical differences (p < 0.05).

The water and the organic matter content in soil was 16.03 ± 5.22 and 4.44 ± 1.26% (mean ± standard error), respectively. The minimum TN content in the soil in the 10th sampling site was 16.56 ± 6.02 mg g^-1^ (mean ± standard error). In the 7th sampling site, the TN content in soil was 27.10 ± 3.81 mg g^-1^, which was the maximum value in the current study. Moreover, no significant difference was observed in the TN content in soils among these sampling sites, and the mean TN content in soil of the salt marsh was 20.59 ± 7.00 mg g^-1^ (Fig 2). The mean TP content in soil in Dongfeng Salt Marsh was 0.31± 0.01 mg g^-1^ (mean ± standard error). The TP content in soil in the 10th sampling site was significantly higher than that in the 9th sampling site (p < 0.05) (Fig 3). Moreover, the ratio of TN/TP in soil in the salt marsh ranged from 40.38 to 102.80, and the mean value was 69.09 ± 5.55 (mean ± standard error).

**Fig 4:**
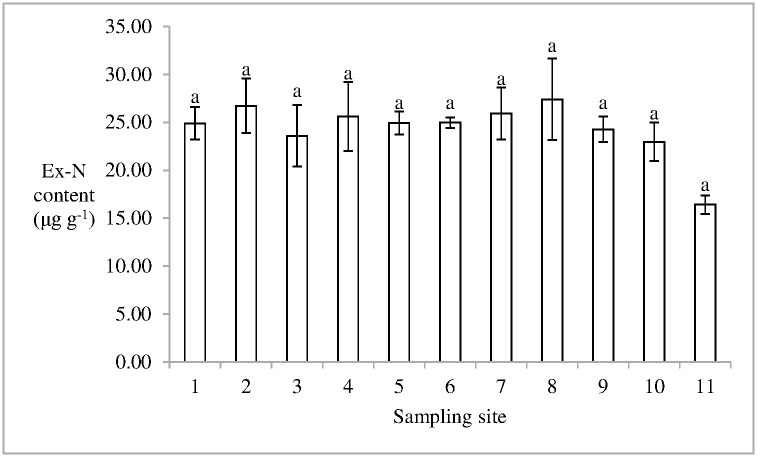
Ex-N content in soil in Dongfeng Salt Marsh. Different letters indicate the statistical differences (p < 0.05).

**Fig 5:**
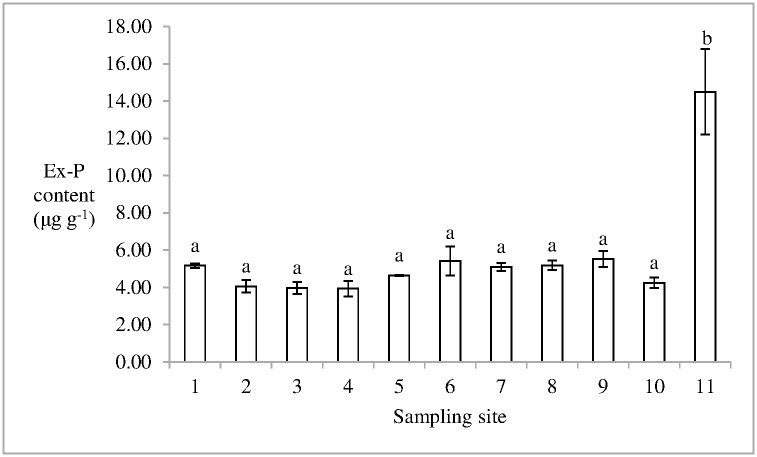
Ex-P content in soil in Dongfeng Salt Marsh. Different letters indicate the statistical differences (p < 0.05).

The ratio of Ex-N/TN in soil in the salt marsh was 0.12±0.01%(mean ± standard error). The Ex-N content in soil in the 11th sampling site was 16.41 ± 0.96 μg g^-1^ (mean ± standard error), which was the minimum content in Dongfeng Salt Marsh. The 8th sampling site had the maximum Ex-N content in soil, 27.40 ± 4.26 μg g^-1^. No significant difference in Ex-N content was found in soil between the two sampling sites, which indicates that significant difference was not observed in the salt marsh (Fig 4). The Ex-P content accounted for 1.84±0.29% of TP content in soils in the salt marsh. The mean Ex-P content in soil in the salt marsh was 5.61 ± 0.54 μg g^-1^ (mean ± standard error). The Ex-P content in soil in the 11th sampling site was higher than those in other sampling sites (p < 0.05) (Fig 5). The ratio of Ex-N/Ex-P in soil in the salt marsh ranged from 1.13 to 6.58, and the mean value was 5.02 ± 0.44 (mean ± standard error).

### Nitrogen and phosphorus contents in plant

**Fig 6:**
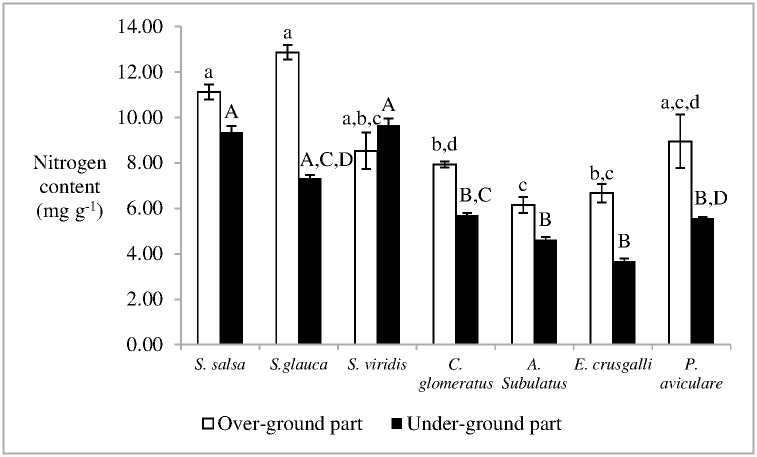
Nitrogen content in plant in Dongfeng Salt Marsh. Different letters indicate the statistical differences (p < 0.05).

The nitrogen content in the over-ground part of *S*. *salsa* was 11.12 ± 0.33 mg g^-1^ (mean ± standard error), which was higher than those of *A*. *subulatus*, *C*. *glomeratus*, and *E*. *Crusgalli* (p < 0.05) (Fig 6). Moreover, the nitrogen content in the underground part of *S*. *salsa* was 9.35 ± 0.26 mg g^-1^, which was higher than those of *A*. *subulatus*, *C*. *glomeratus*, *E*. *crusgalli*, and *P*. *aviculanre* (p < 0.05) (Fig 6).

**Fig 7:**
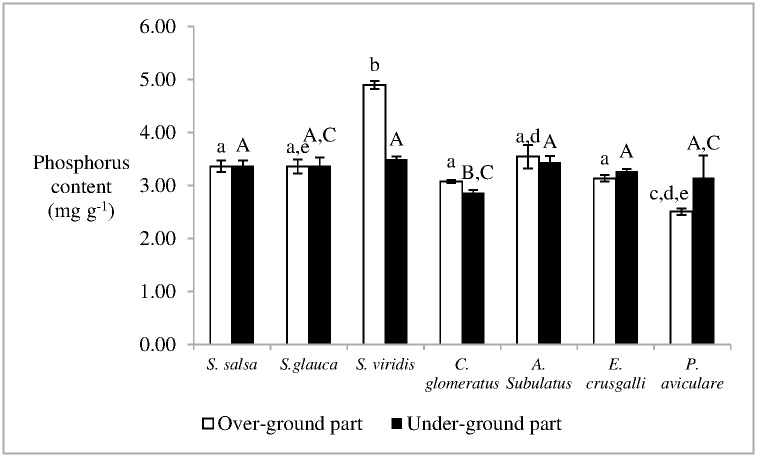
Phosphorus content in plant in Dongfeng Salt Marsh. Different letters indicate the statistical differences (p < 0.05).

The phosphorus content in the over-ground part of *S*. *viridis* was 4.90 ± 0.07 mg g^-1^ (mean ± standard error), which was higher than those of other plants in the salt marsh (p < 0.05). Moreover, the phosphorus content in the over-ground part of *P*. *aviculare* was 2.51 ± 0.06 mg g^-1^, which was less than those of *S*. *salsa*, *S*. *viridis*, *E*. *crusgalli*, and *C*. *glomeratus* (p < 0.05) (Fig 7). The phosphorus content in the underground part of *C*. *glomeratus* was 2.87 ± 0.05 mg g^-1^, which was less than those of *S*. *salsa*, *S*. *viridis*, *E*. *crusgalli*, and *A*. *subulatus* (p < 0.05) (Fig 7).

**Fig 8:**
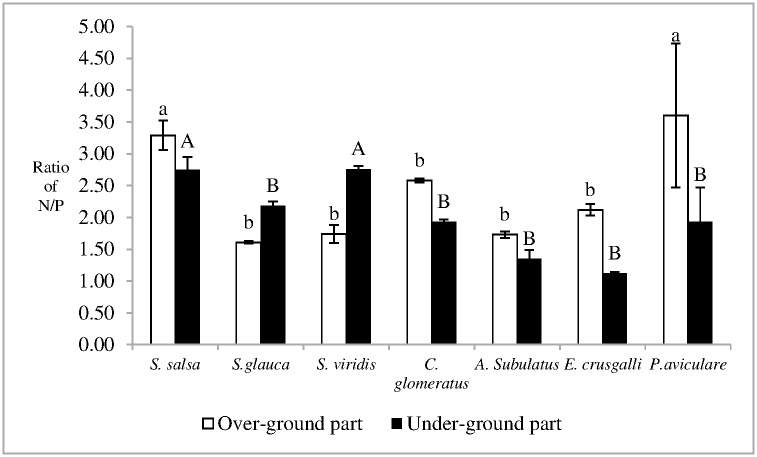
Ratio of N/P in plant in Dongfeng Salt Marsh. Different litters indicate the statistical differences (p < 0.05).

Significant difference in the ratio of nitrogen/phosphorus was not found between over-ground parts of *S*. *salsa* and *P*. *aviculare*, which were 3.29 ± 0.23 and 3.60 ± 1.13, respectively. The ratio of nitrogen/phosphorus in the over-ground part of *S*. *salsa* was significantly higher than those of *E*. *crusgalli*, *C*. *glomeratus*, *S*. *viridis*, *A*. *subulatus*, and *S*. *glauca* (p < 0.05) (Fig 8). The ratio of nitrogen/phosphorus of the underground part of *E*. *crusgalli*, *C*. *glomeratus*, *A*. *subulatus*, *P*. *aviculare*, and *S*. *glauca* was less than that of *S*. *salsa* or *S*. *viridis* (p < 0.05) (Fig 8). The ratio of nitrogen/phosphorus of the underground part of *S*. *salsa* was similar to that of *S*. *viridis*, which was 2.75 ± 0.20 and 2.76 ± 0.05, respectively.

## Discussion

### High colonization possibility of invasive weeds in *S*. *salsa* community

Vegetation types had significant effects on soil nutrients and soil ecological stoichiometry characteristics [21]. There was 6 types of vegetations in the study area, hence, the TN, Ex-N, TP and Ex-P contents in soils varied a lot in Dongfeng Salt Marsh. Moreover, the ratio of nitrogen/phosphorus in soil is about 43 if the soil is mainly influenced by natural ecological process, whereas this ratio is 12 if the soil is mainly influenced by anthropogenic activitieswhich may the ratio of nitrogen/phosphorus in soil [22]. The ratio of TN/TP in soil in Dongfeng Salt Marsh ranged from 40.38 to 102.80. Thus, the salt marsh was less influenced by anthropogenic activities than natural processes, which implied that the fragmentation of *S*. *salsa* communities in the salt marsh was influenced more by natural processes. Thus, the mechanisms of invasion and colonization of weeds in *S*. *salsa* communities may be explained from an ecological stochiometric perspective.

Phytoplankton absorbs nutrients such as nitrogen and phosphorus at a specific ratio, which indicates that the Sheffield ratio can be used to evaluated relative nutrient limitation in water areas [23]. If the ratio of nitrogen/phosphorus in soil is larger than 13, there may be unequivocal nitrogen limitation for primary production [24]. Threshold values for unequivocal phosphorus limitation are debatable, and vary from 16 to 20; these thresholds depend on plant growth and habitat, although 16 remains apparently suitably for most herbaceous communities in wetlands [25]. For example, if terrestrial plants are limited by phosphorus, the ratio of nitrogen/phosphorus in soil may be larger than 16. The ratio of TN/TP in soil in Dongfeng Salt Marsh was larger than 16, which implies that plant growth may be limited by phosphorus in the salt marsh. Nevertheless, the TN in soil includes exchangeable nitrogen, weak acid-extractable nitrogen, strong alkaili-extractable nitrogen, strong oxidant-extractable nitrogen, non-extractable nitrogen, and organic nitrogen [26]. The TP in soil includes exchangeable phosphorus, aluminum-bound phosphorus, iron-bound phosphorus, occluded phosphorus, authigenic calcium phosphorus, detrital phosphorus, and organic phosphorus [27]. The survival and growth of plants are influenced by the masses and forms of nutrients [28–30]. Organic nitrogen may be directly used by plants in wetlands [31]. Nevertheless, inorganic nitrogen or inorganic phosphorus is the dominant composition of nutrients that can be directly used by plants. Moreover, the exchangeable nitrogen (phosphorus) has the highest biological activity, and some forms of nitrogen (phosphorus) such as residual state nitrogen (phosphorus) can’t be used by plants effectively. Thus, using the ratio of TN/TP in soil to evaluate nutrient limitation for primary production is not suitable. The nutrient availability in soil determines the utilization of plant nutrients [32], which indicates that the ratio of Ex-N/Ex-P is suitable to be used to assess nutrient limitation in soil. The ratio of Ex-N/Ex-P in soil in Dongfeng Salt Marsh was less than 13, which indicated that a relative nitrogen limitation exists from an ecological stochiometric perspective of soil in the salt marsh.

The ecological stoichiometric characteristics of plants especially the leaf stoichimentric characteristics are used to evaluate nutrient limitations [33,34]. If the ratio of nitrogen/phosphorus in plants is less than 14, a relative nitrogen limitation for plants may exist [35]. There's no significant difference of nitrogen contents, phosphorus contents and nitrogen/phosphorus in leaves, stems and above organs of wetland vegetations across China [36]. The ratio of nitrogen/phosphorus of root or over-ground part can be used to evaluate nutrient limitation [21, 37]. Moreover, the leaf, flower and fruit of *S*. *salsa* can't be differentiate during the sapling period. Thus, ratio of nitrogen/phosphorus of over-ground and underground parts of plants was used to assess the nutrient limitation in the present work. The maximum ratios of nitrogen/phosphorus of over-ground and underground parts of plants in Dongfeng Salt Marsh were 3.60 ± 1.13 and 2.76 ± 0.05, respectively. Thus, a relative nitrogen limitation for plants may occur in the salt marsh.

Absolute phosphorus limitation for plants exists if the phosphorus content in plants is less than 0.7 mg g^-1^ or 1 mg g^-1^ [25, 38]. The minimum phosphorus contents of phosphorus contents of over-ground and underground parts of plants in Dongfeng Salt Marsh were 2.51 ± 0.06 mg g^-1^ and 2.87 ± 0.05 mg g^-1^, respectively. Thus, the phosphorus in the salt marsh may be abundant for plants. The mean nitrogen content of terrestrial plants is approximately 20.6 mg g^-1^ [39], and it is 20.2 mg g^-1^ in the mainland of China [40]. Moreover, absolute nitrogen limitations for plants may occur if the nitrogen content in plants ranges from 13 to 14 mg g^-1^ [38]. The maximum nitrogen content of the over-ground part of plants in Dongfeng Salt Marsh was 12.87 ± 0.32 mg g^-1^ and that of the underground part of plants was 9.67 ± 0.30 mg g^-1^. Given these results, absolute nitrogen limitation for plants occurred in the salt marsh, which accorded with the stoichiometric characteristics of almost all wetlands in the mainland of China, where 98% of all wetlands were limited by nitrogen [36]. In Yancheng coastal wetland, Jiangsu Province, China, *S*. *salsa* community was limited by nitrogen [41]. The nitrogen limitation may be caused by differences of nitrogen and phosphorus biogeochemical process. Salinity limits the availability of mineral nutrients in soil of coastal wetland. Organic nitrogen mineralization belongs to one biological process, whereas phosphorus weathering belongs to one chemical process mainly, which is less sensitive to salinity.

Runoffs exist in coastal wetlands, which are the main habitats for birds feed on plant seeds. The wind velocity in coastal zones is usually fast [42]. These phenomena lead to a high possibility of different plant seed banks in the coastal saline wetlands. With the high possibility of germination and survival of *S*. *salsa* under adverse conditions [43], the bare land may be covered by *S*. *salsa*, which can provide relatively favorable conditions for other plants by reducing soil salinity or alkalinity [7]. Thus, seed germination of invaded plants in the *S*. *salsa* community is a high possibility. Plants with low nitrogen content adapt nitrogen limitation soil generally [44]. Given the relatively low nitrogen content in *C*. *glomeratus*, *E*. *crusgalli*, and *A*. *subulatus*, these species needed less nitrogen to grow and breed. These species exhibited higher competitiveness than *S*. *salsa*, which indicates that they may have a high capacity to colonize in *S*. *salsa* community because the plants in the salt marsh were limited by nitrogen. Nevertheless, the colonization of *S*. *viridis*, *P*. *aviculare* and *S*. *glauca* in *S*. *salsa* community may be accidental, because no significant difference in nitrogen contents between these plants and *S*. *salsa* was found.

### Implication for *S*. *salsa* community succession

Botany invasion and successful colonization are the basis of community succession. Invasion and colonization of *C*. *glomeratus*, *E*. *crusgaill*, and *A*. *subulatus* in *S*. *salsa* communities lead to fragmentation in *S*. *salsa* communities. In the Yellow River Delta, the positive *S*. *salsa* community starts from *S*. *salsa* to *Gramineous* plants [45], during which *S*. *salsa* was replaced by *Gramineous* plants, and the *S*. *salsa* community declined. Thus, the *S*. *salsa* community fragmentation caused by *C*. *glomeratus*, *E*. *crusgaill*, and *A*. *subulatus* colonization isnot a degradation of *S*. *salsa* wetland, but a positive community succession trend of *S*. *salsa* community. In Dongfeng Salt Marsh, the plant growth was limited by nitrogen. The nitrogen content in *S*. *salsa* was higher than those in *C*. *glomeratus*, *E*. *crusgalli*, and *A*. *subulatus*, and it was similar to those in *S*. *glauca* and *S*. *viridis*. The *S*. *salsa* population in Dongfeng Salt Marsh may be replaced by *C*. *glomeratus*, *E*. *crusgalli*, and *A*. *subulatus* if the current situation of the salt marsh is maintained. In addition, *S*. *glauca* and *S*. *viridis* may be companion species of *S*. *salsa*, and the *S*. *salsa* community may not be replaced by *S*. *glauca* and *S*. *viridis*. Because the nitrogen content in the underground part of *P*. *aiculare* was less than that of *S*. *salsa* and no significant difference in the nitrogen content of the over-ground part was observed between *P*. *aviculare* and *S*. *salsa*, there must be another way for *P*. *aviculare* to use nitrogen. Thus, *P*. *aviculare* tends to replace the *S*. *salsa* community in Dongfeng Salt Marsh.

The colonization of *Gramineous* or *Compositae* plants in *S*. *salsa* community may reduce their sightseeing values. Nitrogen addition may decrease biodiversity in nitrogen limitation zones [46]. Thus, nitrogen fertilization may be an effective method to increase the growth rate of *S*. *salsa*. Moreover, *S*. *salsa* tends to accelerate growth stem and branch in supratidal zone [47], which means there would be more *S*. *salsa* litters if the growth rate of *S*. *salsa* is increased in supratidal zone. Salinity in soil is absorbed by *S*. *salsa* effectively [48], and salinity will release into soil during *S*. *salsa* litters decomposition which increases as increased salinity [49]. Thus, if the *S*. *salsa* litters are not transfered from the *S*. *salsa* community, it is difficult for non-halophytes seeds to germinate in zones covered with superfluous *S*. *salsa* litters. *S*. *salsa* is one halophytes species and its seeds germination are less influenced by salinity [1]. Nevertheless, *C*. *glomeratus*, *E*. *crusgalli*, *A*. *subulatus*, and *P*. *aviculare* belong to non-halophytes. The Dongfeng Salt Marsh is located in supratidal zoneand the *S*. *salsa* isn't be harvested in the wetland. Thus, nitrogen fertilization may be effective to maintain *S*. *salsa* community in Donfeng Salt Marsh, because seedsgermination of *C*. *glomeratus*, *E*. *crusgalli*, *A*. *subulatus*, and *P*. *aviculare* that colonized in *S*. *salsa* community would be restrained by superfluous *S*. *salsa* litters if the biological available nitrogen supply is sufficiently available. Moreover, water content and salinity in soil may be the most important impact factors for *S*. *salsa* community succession in coastal wetlands [50], effects of nitrogen fertilization on controlling weeds invasion and colonization in *S*. *salsa* community needs more studies to verify.

## Supporting Information

S1 Fig This is the Fig 2. This is the TN content in soil in Dongfeng Salt Marsh.
(PDF)

S2 Fig This is the Fig 3. This is the TP content in soil in Dongfeng Salt Marsh.
(PDF)

S3 Fig This is the Fig 4. This is the Ex-N content in soil in Dongfeng Salt Marsh.
(PDF)

S4 Fig This is the Fig 5. This is the Ex-P content in soil in Dongfeng Salt Marsh.
(PDF)

S5 Fig This is the Fig 6. This is the nitrogen content in plant in Dongfeng Salt Marsh.
(PDF)

S6 Fig This is the Fig 7. This is the phosphorus content in plant in Dongfeng Salt Marsh.
(PDF)

S7 Fig This is the Fig 8. This is the ratio of N/P in plant in Dongfeng Salt Marsh.
(PDF)
