# Supplementary figures and images for: High Colonization Possibility of Some Species of Weeds in Suaeda salsa Community: From an Ecological Stoichiometry Perspective
Source: PLoS One. 2017 Jan 30;12(1):e0170401. doi: 10.1371/journal.pone.0170401 (PMC5279750; doi:10.1371/journal.pone.0170401)

|      | Sampling mean | standard error |
|------|---------------|----------------|
| 1st  | 17.09         | 4.52           |
| 2nd  | 21.03         | 7.00           |
| 3rd  | 23.36         | 0.95           |
| 4th  | 20.66         | 3.10           |
| 5th  | 17.39         | 3.78           |
| 6th  | 25.04         | 1.32           |
| 7th  | 27.10         | 3.81           |
| 8th  | 22.84         | 4.02           |
| 9th  | 19.62         | 3.26           |
| 10th | 16.56         | 6.02           |
| 11th | 15.76         | 4.35           |

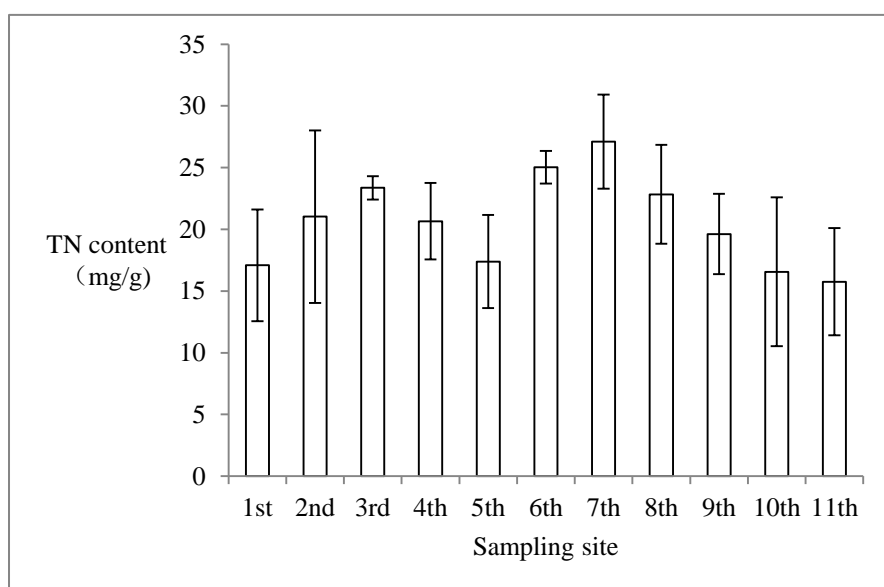

Supplement: S1 Fig — This is the TN content in soil in Dongfeng Salt Marsh. (PDF) [file pone.0170401.s001.pdf]

| Sampling mean | standard error |      |
|---------------|----------------|------|
| 1st           | 0.31           | 0.02 |
| 2nd           | 0.33           | 0.03 |
| 3rd           | 0.35           | 0.01 |
| 4th           | 0.30           | 0.01 |
| 5th           | 0.26           | 0.01 |
| 6th           | 0.35           | 0.02 |
| 7th           | 0.27           | 0.02 |
| 8th           | 0.28           | 0.01 |
| 9th           | 0.25           | 0.02 |
| 10th          | 0.39           | 0.05 |
| 11th          | 0.32           | 0.03 |

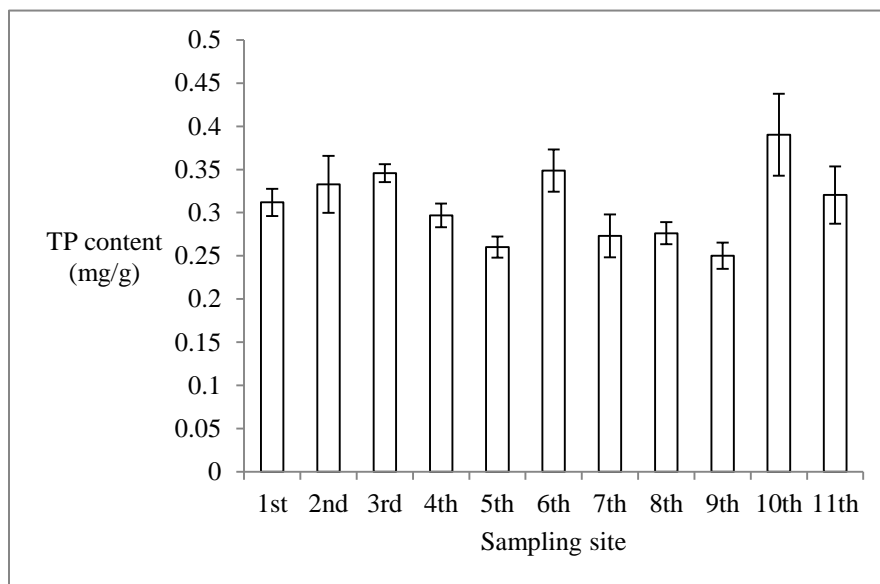

Supplement: S2 Fig — This is the TP content in soil in Dongfeng Salt Marsh. (PDF) [file pone.0170401.s002.pdf]

Sampling mean      standard error

|      |       |      |
|------|-------|------|
| 1st  | 24.90 | 1.70 |
| 2nd  | 26.71 | 2.84 |
| 3rd  | 23.58 | 3.21 |
| 4th  | 25.61 | 3.61 |
| 5th  | 24.95 | 1.20 |
| 6th  | 24.97 | 0.53 |
| 7th  | 25.94 | 2.71 |
| 8th  | 27.40 | 4.26 |
| 9th  | 24.28 | 1.34 |
| 10th | 22.97 | 2.02 |
| 11th | 16.41 | 0.96 |

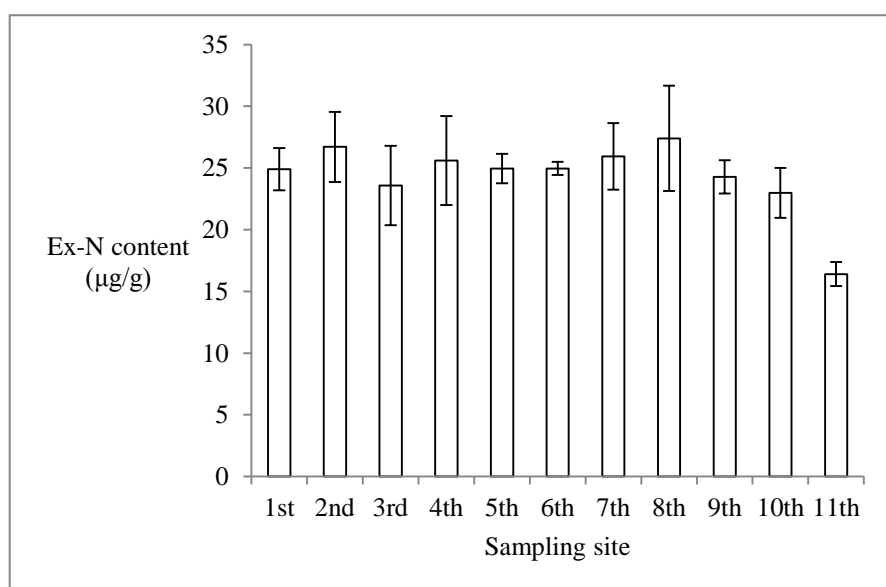

Supplement: S3 Fig — This is the Ex-N content in soil in Dongfeng Salt Marsh. (PDF) [file pone.0170401.s003.pdf]

| Sampling | mean  | standard error |
|----------|-------|----------------|
| 1st      | 5.17  | 0.21           |
| 2nd      | 4.06  | 0.57           |
| 3rd      | 3.97  | 0.55           |
| 4th      | 3.93  | 0.70           |
| 5th      | 4.63  | 0.04           |
| 6th      | 5.41  | 1.35           |
| 7th      | 5.09  | 0.38           |
| 8th      | 5.19  | 0.45           |
| 9th      | 5.53  | 0.73           |
| 10th     | 4.25  | 0.49           |
| 11th     | 14.50 | 3.98           |

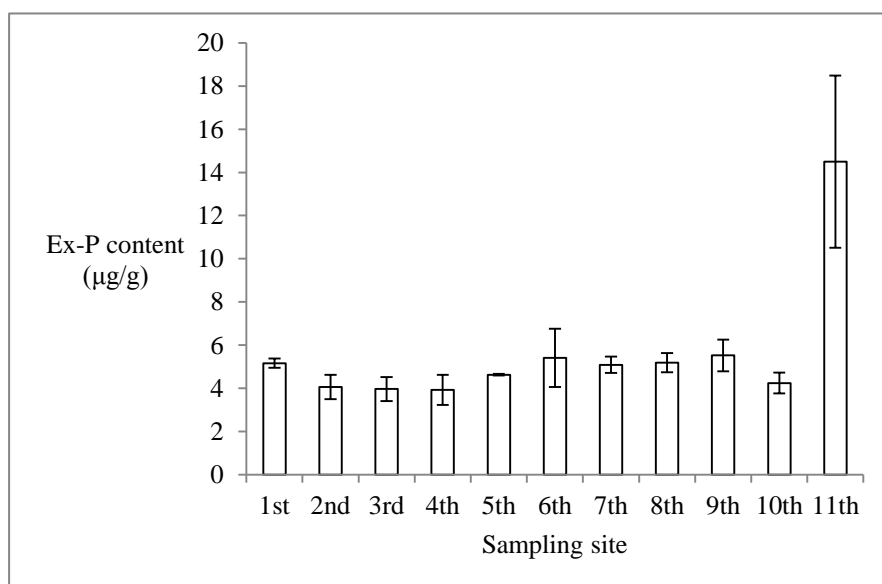

Supplement: S4 Fig — This is the Ex-P content in soil in Dongfeng Salt Marsh. (PDF) [file pone.0170401.s004.pdf]
