# Supplementary material for: High Colonization Possibility of Some Species of Weeds in Suaeda salsa Community: From an Ecological Stoichiometry Perspective
Source: PLoS One. 2017 Jan 30;12(1):e0170401. doi: 10.1371/journal.pone.0170401 (PMC5279750; doi:10.1371/journal.pone.0170401)

	above ground part		under-ground part	
Weed spe	mean	standard	mean	standard error
S. salsa	3.36	0.11	3.38	0.10
S.glauca	3.36	0.13	3.38	0.15
S. viridis	4.90	0.07	3.50	0.05
C. glome	3.08	0.03	2.87	0.05
A. Subula	3.55	0.22	3.44	0.11
E. crusga	3.14	0.06	3.27	0.05
P. avicula	2.51	0.06	3.15	0.42

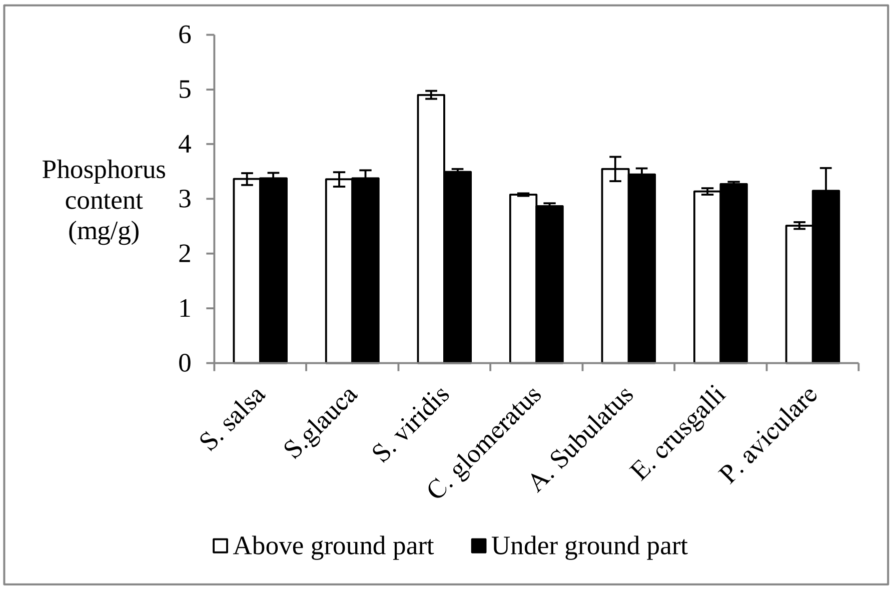

Supplement: S6 Fig — This is the phosphorus content in plant in Dongfeng Salt Marsh. (PDF) [file pone.0170401.s006.pdf]
